# Establishment of National Diagnostic Reference Levels 2025 for nuclear medicine in Japan

**DOI:** 10.1007/s12149-025-02102-y

**Published:** 2025-09-03

**Authors:** Koichiro Abe, Shingo Baba, Reo Etani, Takahiro Fujimto, Makoto Hosono, Takashi Iimori, Anri Inaki, Masanobu Ishiguro, Noriaki Miyaji, Atsutaka Okizaki, Takeshi Sasaki, Hiroyuki Tsushima, Hiroshi Watanabe, Masanori Watanabe, Nobuhiro Yada

**Affiliations:** 1 Department of Radiology, Tokyo Medical University, 6-7-1 Nishishinjuku, Shinjuku-Ku, Tokyo, 160-0023 Japan; 2 Department of Health Sciences, Graduate School of Medical Sciences, Kyushu University, 3-1-1 Maidashi, Higashi-Ku, Fukuoka, 812-8582 Japan; 3 Department of Environmental Health Science, Oita University of Nursing and Health Sciences, 2944-9 Megusuno, Oita, 870-1201 Japan; 4 Division of Clinical Radiology Service, Kyoto University Hospital, 54 Shogoin-Kawahara-Cho, Sakyo-Ku, Kyoto, 606-8507 Japan; 5 Department of Radiology, Faculty of Medicine, Kindai University, 377-2 Ohno-Higashi, Osaka, Osaka-Sayama 589-8511 Japan; 6 Department of Radiology, Chiba University Hospital, 1-8-1 Inohana, Chuo-Ku, Chiba, 260-8677 Japan; 7 Division of Functional Imaging, Exploratory Oncology Research and Clinical Trial Center, National Cancer Center, 6-5-1 Kashiwanoha, Kashiwa, Chiba 277-8577 Japan; 8 Section of Radiology, Fujita Health University Hospital, 1-98 Dengakugakubo, Kutsukake-Cho, Toyoake, Aichi 470-1192 Japan; 9 Department of Radiological Sciences, School of Health Sciences, Fukushima Medical University, 10-6 Sakaemachi, Fukushima, 960-8516 Japan; 10 Department of Radiology, Asahikawa Medical University, 2-1-1-1 Midorigaoka-Higashi, Asahikawa, Hokkaido 078-8510 Japan; 11 Department of Radiologic Technology, Ageo Central General Hospital, 1-10-10 Kashiwaza, Ageo, Saitama 362-8588 Japan; 12 Department of Radiological Technology, Kobe Tokiwa University, 2-6-2 Otanicho, Nagata-Ku, Kobe, Hyogo 653-0838 Japan; 13 Department of Radiological Sciences, Gunma Paz University, 1-7-1 Tonya-Machi, Takasaki, Gunma 370-0006 Japan; 14 Department of Radiology, Shimane University Hospital, 89-1 Enya-Cho, Izumo, Shimane 693-8501 Japan

**Keywords:** Diagnostic reference level, National DRL, Nuclear medicine, Radiopharmaceutical, Hybrid CT

## Abstract

Diagnostic reference levels (DRLs) are practical benchmarks for optimizing patient radiation exposure in medical imaging. In Japan, national DRLs, including those for nuclear medicine together and other radiological procedures, were first established in 2015 and revised in 2020. In this study, we revised the DRL values of nuclear medicine for the establishment of DRLs2025, based on data collected from institutions nationwide throughout Japan. Data were collected via an online survey from facilities performing nuclear medicine procedures, including SPECT, PET, and hybrid CT imaging. Information on dose activity of the administered radiopharmaceuticals and CT parameters (CTDIvol and DLP) were collected. DRL values were determined through analysis of the submitted data, supplemented by panel discussions among experts taking into account the clinical appropriateness of the values and various technological factors. Overall, the newly established DRLs2025 demonstrated a decreasing trend in administered radiopharmaceutical activities, CTDIvol, and DLP compared with the previous surveys. This trend reflects ongoing efforts toward the optimization of radiation exposure and radiopharmaceutical dose reduction, likely driven by the introduction of image reconstruction methods based on newer technologies. However, substantial interfacility variations were observed, particularly in the CT parameters, suggesting disparities in equipment, imaging protocols, and the balance between image quality and radiation dose. The establishment of DRLs2025 underscores continued progress in optimizing radiation exposure in nuclear medicine practice in Japan. Although issues regarding data variability and quality remain, DRLs continue to be a key tool in radiation protection and quality assurance. Ongoing efforts to improve data collection systems and to align procedures with international standards are essential for the future refinement of DRLs.

## Introduction

Diagnostic reference levels (DRLs) are essential tools for the optimization of radiation exposure in medical imaging. They provide benchmarks for typical dose levels associated with standard imaging procedures, and help identify instances in which radiation doses are unusually high or low. In Japan, the first national DRLs for nuclear medicine were established in 2015 under the initiative of the Japan Network for Research and Information on Medical Exposure (J-RIME), based on a nationwide survey of administered radiopharmaceutical activities and imaging practices, and the results were published in 2016 [1]. This pioneering effort laid the foundation for systematic radiation dose optimization in nuclear medicine throughout Japan.

Building on this work, J-RIME coordinated a second nationwide survey and the revised national DRLs were published in 2020 [2]. This revision not only updated the reference levels for radiopharmaceutical administration doses, but also, for the first time, established DRLs for hybrid computed tomography (CT), namely CT components of single-photon emission computed tomography (SPECT)/CT and positron emission tomography (PET)/CT. This addition reflected the growing clinical use of hybrid imaging procedures, and recognized the importance of optimizing radiation exposure from the CT portion, which contributes substantially to the overall patient radiation dose in the nuclear medicine field.

In recent years, the importance of radiation dose optimization has been further underscored by emerging lines of evidence regarding the potential risks associated with medical imaging. A recent study published in *JAMA Internal Medicine* reported that approximately 103,000 future cancers could result from CT examinations performed in the United States in 2023 [3], highlighting the need for continued efforts to minimize unnecessary radiation exposure. Such findings emphasize the crucial role of DRLs in guiding clinical practice to balance diagnostic benefits with radiation risks, and hybrid CT that accompanies PET and SPECT is no exception, albeit being low-dose CT.

The International Commission on Radiologic Protection [4] and International Atomic Energy Agency [5] recommend that DRLs be reviewed periodically in response to social conditions surrounding healthcare, such as technological innovations in medical devices and the development of new radiopharmaceuticals. In response to ongoing technological advancements, changes in diagnostic protocols, and increasing awareness of radiation protection, J-RIME has conducted its third comprehensive nationwide survey to collect updated data on administered radiopharmaceutical activities and CT parameters across Japan. This paper presents the results of that survey and proposes revised DRLs2025 based on current clinical practices, aiming to support ongoing optimization efforts in Japan.

## Materials and methods

### National survey methods

A nationwide survey was conducted in Japan to collect data on the dose activity of radiopharmaceuticals and the dose data from hybrid CT of SPECT/CT and PET/CT in nuclear medicine imaging studies. The methodology followed was essentially based on those used in previous national surveys reported in 2016 [1] and 2020 [2], with minor modifications to reflect updates in imaging protocols and equipment.

Prior to conducting the questionnaire survey, a document explaining the outline of the survey was sent to the heads of nuclear medicine facilities, on-site staff, and radiation safety managers throughout Japan. In addition, participation in the survey was requested through relevant professional societies. Then the survey was implemented in the form of internet-based questions to nuclear medicine facilities throughout Japan, covering a wide range of facilities, including university hospitals, public and private hospitals, and diagnostic imaging centers. The survey requested information on commonly performed diagnostic nuclear medicine procedures in adult patients, including hybrid imaging modalities, such as SPECT/CT and PET/CT. For each procedure, respondents were asked to provide the median actual administered activity (MBq), and the patient exposure parameters, namely volume computed tomography dose index (CTDIvol, mGy) and dose-length product (DLP, mGy·cm), as displayed on the hybrid CT console. The CTDIvol and DLP values were calculated based on measurements using International Electrotechnical Commission (IEC) CT dosimetry phantom. Typically, the 16-cm phantom is used for head scans, whereas the 32-cm phantom is used for body scans, including the head and neck region.

Responses were anonymized and consolidated for analysis. In principle, the survey covered nuclear medicine examinations performed during July 2024, with the period extended for rare examinations. As in the previous survey, the survey items were nuclear medicine tests that were covered by insurance in Japan at the time of the survey.

This work was exempted from ethical review because the data collected were only median values for all responding institutions, and did not include personal information. In addition, because individual data for each institution would not be published, there was no need for individual responding institutions to obtain ethics committee approval.

### Establishment of DRLs for radiopharmaceuticals

Each site provided via the internet the median radioactivity administered to a standard adult patient for each nuclear medicine examination. Data from each facility were combined for each nuclear medicine test, and the 50th and 75th percentile values were calculated after outliers were excluded. The final DRL values were determined by consensus, without necessarily sticking to the 75th percentile values, and were expressed using no more than two integers for easy recognition at a glance. In determining Diagnostic Reference Levels 2025 (DRL2025), as the amount of radiopharmaceutical administered directly affects image quality in nuclear medicine examinations, the focus was placed on ensuring sufficient image quality for diagnosis, and to avoid setting excessively low values. In cases in which data were insufficient or inconsistent, values were supplemented by referring to DRLs2015 and DRLs2020, accompanying documents, or clinical guidelines. In addition, when different radiopharmaceuticals were used for the same target organ, DRLs were set to the same value, or as close as possible if the isotopes were the same (e.g., HMPAO and ECD in cerebral blood flow scintigraphy, and MDP and HMDP in bone scintigraphy). As in the previous survey, pediatric doses were not included in this survey, as the “Japanese Consensus Guidelines for the Optimization of Pediatric Nuclear Medicine Examinations [6]” revised in 2020 should be followed for pediatric patients.

### Establishment of DRLs for hybrid CTs

The facilities were also asked to report median CTDIvol and DLP values for hybrid CT of SPECT/CT and PET/CT. With the increase in the use of SPECT/CT and PET/CT in recent decades, the role of hybrid CT in nuclear medicine appears to be shifting from its original purpose of simple attenuation correction only to include a diagnostic component with additional information from CT. Therefore, the distinction between the terms “attenuation correction (AC)” and “diagnosis (Dx)” used in DRLs 2020 is no longer clear in clinical practice, and to avoid confusion among survey respondents, the questionnaire was revised to avoid these terms as much as possible. That is, “AC” was limited to brain and heart examinations only, and examinations of the trunk/extremities are generally interpreted as being performed for the purpose of “AC + Dx”. As with radiopharmaceuticals, the median CTDIvol and DLP data submitted by each site were compiled, and the 50th and 75th percentile values were calculated for each nuclear medicine examination. The DRLs 2020 were followed for items with a small number of responses, and the final DRL values were expressed using no more than two integers, as in the case of radiopharmaceuticals. As in the previous survey, CT scans performed for diagnostic purposes during nuclear medicine examinations (e.g., chest CT scans performed separately from PET/CT scans) were excluded from the survey.

## Results and discussion

### Response rate

This survey included 1178 nuclear medicine facilities throughout Japan. Of these, representatives from 476 facilities responded to the questionnaire that was posted on a dedicated website on the internet. Thus, the response rate of this survey was 40.4%, which was a substantial increase from the rate of DRL2020 (28%), and almost the same as DRL2015 (41%).

The response rate for the previous DRLs2020 survey was low (28%) [2]. This was partly owing to the fact that the survey was conducted at a time when Japan’s ethical guidelines in medical research were undergoing major revisions, and each facility decided to withhold their responses from the perspective of the protection of personal information. It is clear that the number of responses greatly affects the establishment of reliable DRLs, and we should consider ways to increase the response rate by conducting more thorough publicity activities, and making the questionnaire text as easy to understand as possible.

### DRLs for radiopharmaceuticals

Since the publication of DRLs2020, the following radiopharmaceuticals have been newly covered by insurance in Japan, and were added as new survey items for DRLs2025: ^99m^Tc-PYP and ^99m^Tc-HMDP for cardiac amyloidosis, ^99m^Tc-phytate for sentinel lymph node mapping in uterine cervical, vulvar, and head and neck cancers, and ^18^F-fluciclovine for malignant glioma. We added ^99m^Tc-gas for lung ventilation and ^99m^Tc-HSA-D for testicular scintigraphy, which were missing in the previous survey. In addition, the previous myocardial perfusion item, ^201^Tl-chloride, which did not distinguish between rest and stress, was divided into ^201^Tl-chloride rest, ^201^Tl-chloride stress, and ^201^Tl-chloride rest and stress. The survey results (50th and 75th percentiles), and the determined DRLs2025 for each item are shown in Table 1 (radiopharmaceuticals for planar and SPECT imaging) and Table 2 (radiopharmaceuticals for PET imaging).

**Table 1:** Radiopharmaceuticals for planar and SPECT imaging

Radiopharmaceutical	P50 (MBq)	P75 (MBq)	DRLs2025 (MBq)
Bone: ^99m^Tc-MDP	878	948	930
Bone: ^99m^Tc-HMDP	868	932	930
Bone marrow: ^111^In-chloride	76	80	80
Cerebral blood flow: ^99m^Tc-HMPAO (rest or stress)	740	783	800
Cerebral blood flow: ^99m^Tc-HMPAO (rest and stress)	1200	1200	1200
Cerebral blood flow: ^99m^Tc-ECD (rest or stress)	735	784	800
Cerebral blood flow: ^99m^Tc-ECD (rest and stress)	778	922	1100
Cerebral blood flow: ^123^I-IMP (rest or stress)	182	191	200
Cerebral blood flow: ^123^I-IMP (rest and stress)	249	271	270
Brain receptors: ^123^I-iomazenil	185	195	200
Striatum: ^123^I-ioflupane	181	189	190
Cisternography: ^111^In-DTPA	37	38	40
Thyroid uptake: Na^123^I	8	8.66	10
Thyroid: ^99m^TcO_4_^−^	199	238	240
Parathyroid: ^201^Tl-chloride	76	111	120
Parathyroid: ^99m^TcO_4_^−^	241	300	300
Parathyroid: ^99m^Tc-MIBI	720	802	800
Lung ventilation: ^81m^Kr-gas	185	185	190
Lung ventilation: ^99m^Tc-gas	370	750	750
Pulmonary blood flow: ^99m^Tc-MAA	213	260	260
Radionuclide venography: ^99m^Tc-MAA	422	500	500
Liver/spleen: ^99m^Tc-phytate	185	189	190
Hepatic function: ^99m^Tc-GSA	233	253	260
Hepatobiliary: ^99m^Tc-PMT	218	242	260
Liver and spleen: ^99m^Tc-Sn colloid	140	180	180
Myocardial perfusion: ^201^Tl-chloride (rest)	113	114	120
Myocardial perfusion: ^201^Tl-chloride (stress)	113	113	120
Myocardial perfusion: ^201^Tl-chloride (rest and stress)	113	114	120
Myocardial perfusion: ^99m^Tc-tetrofosmin (rest or stress)	739	824	840
Myocardial perfusion: ^99m^Tc-tetrofosmin (rest and stress)	1073	1180	1200
Myocardial perfusion: ^99m^Tc-MIBI (rest or stress)	740	840	840
Myocardial perfusion: ^99m^Tc-MIBI (rest and stress)	1074	1147	1200
Myocardial fatty acid metabolism: ^123^I-BMIPP	125	130	130
Cardiac sympathetic function: ^123^I-MIBG	124	129	130
Cardiac blood pool: ^99m^Tc-HSA-D	892	932	970
Myocardial infarction: ^99m^Tc-PYP	740	838	840
Cardiac amyloidosis: ^99m^Tc-PYP	740	793	840
Cardiac amyloidosis: ^99m^Tc-HMDP	776	865	870
Salivary gland: ^99m^TcO_4_^−^	258	370	370
Meckel’s diverticulum: ^99m^TcO_4_^−^	370	406	440
Gastrointestinal bleeding: ^99m^Tc-HSA-D	932	1000	1000
Protein leakage: ^99m^Tc-HSA-D	929	1000	1000
Static renal imaging: ^99m^Tc-DMSA	185	204	210
Dynamic renal imaging: ^99m^Tc-MAG3	300	381	380
Dynamic renal imaging: ^99m^Tc-DTPA	352	382	380
Adrenal cortex: ^131^I-adosterol	37	40	40
Adrenal medulla: ^123^I-MIBG	127	130	130
Testis: ^99m^Tc-HSA-D	813	846	850
Tumor: ^201^Tl-chloride	111	114	120
Tumor and inflammation: ^67^ Ga-citrate	112	114	120
Somatostatin receptor: ^111^In-pentetreotide	141	175	220
Lymphatic vessels: ^99m^Tc-HSA-D	429	818	830
Sentinel lymph node (breast cancer): ^99m^Tc-Sn colloid	69	90	120
Sentinel lymph node (breast cancer): ^99m^Tc-phytate	68	88	120
Sentinel lymph node (melanoma): ^99m^Tc-Sn colloid	76	103	120
Sentinel lymph node (melanoma): ^99m^Tc-phytate	91	120	120
Sentinel lymph node (endometrial cancer): ^99m^Tc-phytate	81	87	120
Sentinel lymph node (cervical cancer): ^99m^Tc-phytate	83	98	120
Sentinel lymph node (vulvar cancer): ^99m^Tc-phytate	91	111	120
Sentinel lymph node (head and neck cancer): ^99m^Tc-phytate	90	112	120
RI angiography: ^99m^Tc-HSA-D	932	1000	1000

P50, 50th percentile; P75, 75th percentile

**Table 2:** Radiopharmaceuticals for PET imaging

Radiopharmaceutical	P50 (MBq)	P75 (MBq)	DRL2025 (MBq)
Brain function: C^15^O_2_-gas (2D acquisition)	8000	8000	8000
Brain function: ^15^O_2_-gas (2D acquisition)	6000	6000	6000
Brain function: C^15^O-gas (2D acquisition)	3000	3000	3000
Brain function: C^15^O_2_-gas (3D acquisition)	1650	2250	1800
Brain function: ^15^O_2_-gas (3D acquisition)	3200	4500	4500
Brain function: C^15^O-gas (3D acquisition)	2150	3150	3600
Amyloid: ^18^F-flutemetamol (in-house preparation)	243	272	260
Amyloid: ^18^F-flutemetamol (delivery)	238	257	260
Amyloid: ^18^F-florbetapir (in-house preparation)	373	374	370
Amyloid: ^18^F-florbetapir (delivery)	380	407	370
Amyloid: ^18^F-florbetaben (in-house preparation)	300	305	300
Cerebral glucose metabolism: ^18^F-FDG (in-house preparation)	189	233	230
Cerebral glucose metabolism: ^18^F-FDG (delivery)	249	265	230
Cerebral glucose metabolism: ^18^F-FDG (dose per body weight)	4	4	4
Malignant glioma: ^18^F-fluciclovine (delivery)	225	261	270
Malignant glioma: ^18^F-fluciclovine (dose per body weight)	4	5	5
Myocardial glucose metabolism: ^18^F-FDG (in-house preparation)	227	238	240
Myocardial glucose metabolism: ^18^F-FDG (delivery)	236	274	240
Myocardial glucose metabolism: ^18^F-FDG (dose per body weight)	4	4	4
Myocardial blood flow: ^13^NH_3_ (in-house preparation)	520	592	520
Tumor glucose metabolism: ^18^F-FDG (in-house preparation)	214	229	240
Tumor glucose metabolism: ^18^F-FDG (delivery)	237	261	240
Tumor glucose metabolism: ^18^F-FDG (dose per body weight)	4	4	4
Inflammation*: ^18^F-FDG (in-house preparation)	221	242	240
Inflammation: ^18^F-FDG (delivery)	240	259	240
Inflammation: ^18^F-FDG (dose per body weight)	4	4	4

P50, 50th percentile; P75, 75th percentile

^*^Inflammation refers to large vessel vasculitis and cardiac sarcoidosis

Table 3 (radiopharmaceuticals for scintigraphy) and Table 4 (radiopharmaceuticals for PET) show the extent to which the current DRLs2025 has changed compared with previous DRLs. The percentage change was calculated using the following formula: (DRL2025−DRL2020 or DRL2015)/DRL2020 or DRL2015 × 100. Items showing no change from the previous DRL values and items that were not previously reported as DRLs were excluded. For most radiopharmaceuticals, their values in DRL2025 were equivalent to or lower than those in the previous DRLs, possibly owing to advances in equipment and diagnostic imaging technology, as well as more appropriate optimization of examination protocols. However, DRL values were higher than those in previous surveys for two items, namely ^99m^Tc-PYP for myocardial infarction scintigraphy and ^111^In-pentetreotide for somatostatin receptor scintigraphy.

**Table 3:** Percentage differences between DRL2025 and previous DRLs in SPECT

Radiopharmaceutical	%DRL2020 (%)	%DRL2015 (%)
Bone: ^99m^Tc-MDP	− 2.1	− 2.1
Bone: ^99m^Tc-HMDP	− 2.1	− 2.1
Bone marrow: ^111^In-chloride	0.0	− 33.3
Cerebral blood flow: ^123^I-IMP (rest and stress)	0.0	− 10.0
Cisternography: ^111^In-DTPA	0.0	− 42.9
Thyroid: ^99m^TcO_4_^−^	0.0	− 20.0
Lung ventilation: ^81m^Kr-gas	− 5.0	− 5.0
Liver/spleen: ^99m^Tc-phytate	− 5.0	− 5.0
Myocardial perfusion: ^201^Tl-chloride (rest)	0.0	− 33.3
Myocardial perfusion: ^99m^Tc-tetrofosmin (rest or stress)	0.0	− 6.7
Myocardial perfusion: ^99m^Tc-MIBI (rest or stress)	− 4.5	− 6.7
Cardiac blood pool: ^99m^Tc-HSA-D	0.0	− 3.0
Myocardial infarction: ^99m^Tc-PYP	5.0	5.0
Meckel’s diverticulum: ^99m^TcO_4_^−^	0.0	− 12.0
Gastrointestinal bleeding: ^99m^Tc-HSA-D	− 3.8	− 3.8
Protein leakage: ^99m^Tc-HSA-D	− 3.8	NS
Dynamic renal imaging: ^99m^Tc-MAG3	0.0	− 5.0
Dynamic renal imaging: ^99m^Tc-DTPA	− 2.6	− 5.0
Adrenal cortex: ^131^I-adosterol	0.0	− 9.1
Tumor: ^201^Tl-chloride	0.0	− 33.3
Tumor and inflammation: ^67^ Ga-citrate	0.0	− 40.0
Somatostatin receptor: ^111^In-pentetreotide	83.3	NS
Lymphatic vessels: ^99m^Tc-HSA-D	0.0	− 12.6

*NS*: Not surveyed

**Table 4.** Percentage differences between DRL2025 and previous DRLs in PET

PET radiopharmaceutical	%DRL2020 (%)	%DRL2015 (%)
Brain function: C^15^O_2_-gas (3D acquisition)	0.0	− 37.9
Brain function: ^15^O_2_-gas (3D acquisition)	0.0	− 35.7
Brain function: C^15^O-gas (3D acquisition)	0.0	− 52.0
Cerebral glucose metabolism: ^18^F-FDG (in-house preparation)	− 4.2	− 4.2
Cerebral glucose metabolism: ^18^F-FDG (delivery)	− 4.2	− 4.2
Myocardial glucose metabolism: ^18^F-FDG (dose per body weight)	− 20.0	NS
Myocardial blood flow: ^13^NH_3_ (in-house preparation)	0.0	− 27.8

*NS*: Not surveyed

For myocardial infarction scintigraphy using ^99m^Tc-PYP, the dose was increased by 5%, from 800 MBq in DRL2020 and DRL2015 to 840 MBq in DRL2025. The reason for the increase is not clear, but may simply reflect the higher 75th percentile value in the DRLs2025 than in the previous DRLs. The fact that the number of times these tests are being performed has been decreasing in recent years may also be a factor. For the newly added cardiac amyloidosis scintigraphy using ^99m^Tc-PYP, DRL2025 was also set slightly above the 75th percentile. This was set to the same value as for myocardial infarction scintigraphy because the same radiopharmaceutical was used to evaluate the same target organ (Table 1).

For somatostatin receptor scintigraphy, DRL2025 was revised to 220 MBq, which is a significant increase from DRL2020 (120 MBq). This is thought to be because in Japan, the delivery day of ^111^In-pentetreotide (Wednesday) is before the assay date (Friday), and many facilities probably administer unadjusted ^111^In-pentetreotide on Wednesday to avoid performing the examination on the weekend, so the actual administration dose is about 1.6 times the assay dose. In the previous survey, the dose was determined in consideration of the attached package insert, but this time, image quality and actual conditions used in the clinical setting were prioritized, and we set the DRL value to 220 MBq. This value is already higher than the 75th percentile in this survey, but it is the maximum dose excluding outliers in the actual survey responses. This value is within the dose range (111 to 222 MBq) indicated in the diagnostic guidelines of Japan [7], and is lower than the DRL (237 MBq) in the USA (237 MBq) [8].

The following items had fewer than 10 responses: ^99m^Tc-HMPAO (rest and stress) for cerebral blood flow scintigraphy, ^99m^Tc-HSA-D for testicular scintigraphy (newly added), ^99m^Tc-phytate for sentinel lymph node scintigraphy (uterine and cervical cancer), ^15^O-gas for PET, in-house manufactured radiopharmaceuticals for 3 types of amyloid PET, in-house manufactured ammonia for PET, and ^18^F-fluciclovine for PET (newly added). For these items, DRLs were established based on those from the DRLs 2020 or by referring to published guidelines, drug package inserts, and DRLs for similar procedures.

With a deepening of our understanding of the importance of DRLs in optimizing radiation exposure in the field of nuclear medicine, in recent years, national DRLs are beginning to be established in various countries [9–12]. These DRLs are generally similar among various countries, but when individual examinations are directly compared between countries, some of them show considerable heterogeneity. This is thought to be largely owing to differences in the level of medical care and the state of equipment updates in each country and region, but it is also greatly affected by differences in examination protocols, such as whether the administration dose is based on body weight or a fixed amount, whether it is stress-first or rest-first and whether it is a 1-day method or a multi-day method, as is evident in myocardial perfusion scintigraphy [13]. Nuclear medicine examinations encompass a wide variety of procedures. To support the establishment of DRLs and improve their comparability among countries or regions, further efforts are needed to harmonize and standardize nuclear medicine examination protocols across facilities, regions, and countries.

### DRLs for hybrid CT

Similarly, the survey data (50th and 75th percentiles of CTDIvol and DLP) and the proposed DRLs for 2025 for hybrid CT are summarized in Table 5 (SPECT/CT), Table 6 (PET/CT: medical examination), and Table 7 (PET/CT: medical checkup). Based on the previous survey, the survey items for hybrid CT were modified to make it easier to understand the examination purpose and imaging area. Briefly, the number of SPECT/CT items increased from 9 items across 8 areas in the previous survey to 12 items across 10 areas. The use of hybrid CT for attenuation correction was only limited to the brain and heart. For PET/CT, examinations were classified into medical examination and medical checkup (health screening) purposes. Similar to SPECT/CT, the number of survey items increased from 4 items across 3 imaging areas to 13 items across 10 imaging areas, with more detailed categorization to facilitate responses. Use for attenuation correction only was again limited to the brain and heart. The PET/CT items “Whole body: head to lower extremities,” “Head and neck,” “Chest,” “Upper abdomen,” “Pelvis,” “Upper abdomen to pelvis,” “Chest to pelvis,” and “Extremities” were newly established in this survey. However, as the imaging of these specific regions alone is rarely performed in cancer screening, and the actual number of responses was low, these items were excluded from the PET/CT: medical checkup category. Although the number of responses for brain and cardiac PET/CT in the medical checkup context was also limited, it was presumed that a considerable number of examinations, such as amyloid and ammonia PET, were being performed. Therefore, the same DRL values as those used for PET/CT: medical examination were applied.

**Table 5:** CTDIvol and DLP values for SPECT/CT

Body region	CTDIvol (mGy)			DLP (mGy·cm)		
	P50	P75	DRL2025	P50	P75	DRL2025
Whole body	2.200	3.968	4.0	179.3	305.2	310
Head and neck	3.075	5.408	5.4	110.9	162.5	170
Chest	2.495	4.213	4.2	83.10	125.4	130
Upper abdomen	2.055	4.778	4.8	68.60	125.1	130
Pelvis	2.165	3.060	3.1	76.06	110.0	110
Abdomen, pelvis (upper abdomen to pelvis)	2.020	3.875	3.9	85.58	138.2	140
Head and neck to pelvis	2.523	4.140	4.1	156.2	253.2	260
Extremities	2.290	3.090	3.1	99.10	151.5	160
Brain (attenuation correction only)	6.780	12.21	12	82.50	206.0	210
Brain (attenuation correction and image fusion)	11.64	25.10	25	174.0	368.4	370
Heart (attenuation correction only)	1.610	2.923	2.9	35.01	66.16	70
Heart (attenuation correction and image fusion)	1.985	4.068	4.1	49.75	90.75	90

P50, 50th percentile; P75, 75th percentile

**Table 6:** CTDIvol and DLP values for PET/CT: medical examination

Body region	CTDIvol (mGy)			DLP (mGy)		
	P50	P75	DRL2025	P50	P75	DRL2025
Whole body: head to proximal thighs	4.000	5.370	5.4	410.3	531.5	540
Whole body: head to lower extremities	3.460	5.263	5.3	513.8	717.5	720
Head and neck	2.610	4.195	4.2	66.75	127.4	130
Chest	2.955	4.515	4.5	109.0	150.0	150
Upper abdomen	2.865	4.398	4.4	82.61	138.9	140
Pelvis	2.280	3.150	3.2	80.00	114.0	120
Upper abdomen to pelvis	3.040	4.973	5.0	118.3	214.4	220
Chest to pelvis	3.460	4.420	4.4	205.7	300.0	300
Extremities	1.190	2.735	2.7	80.63	124.0	130
Brain (attenuation correction only)	3.140	10.39	10	73.50	261.4	270
Brain (attenuation correction and image fusion)	12.90	25.92	26	253.7	564.6	570
Heart (attenuation correction only)	3.970	4.650	2.5	97.70	201.2	50
Heart (attenuation correction and image fusion)	3.370	4.730	4.7	83.62	133.1	140

P50, 50th percentile; P75, 75th percentile

**Table 7:** CTDIvol values for PET/CT: medical checkup

Body region	CTDIvol (mGy)			DLP (mGy·cm)		
	P50	P75	DRL2025	P50	P75	DRL2025
Whole body: head to proximal thighs	4.015	5.318	5.4	425.5	527.5	540
Brain (attenuation correction only)	–	–	10	–	–	270
Brain (attenuation correction and image fusion)	16.80	21.59	26	332.0	537.4	570
Heart (attenuation correction only)	–	–	2.5	–	–	50
Heart (attenuation correction and image fusion)	7.650	8.925	4.7	158.8	178.9	140

P50, 50th percentile; P75, 75th percentile; –, could not be calculated owing to a small number of responses

In both SPECT/CT and PET/CT, and for both CTDIvol and DLP, the DRLs2025 demonstrated an overall decreasing trend compared with the previous survey, aligning with the trend observed for radiopharmaceutical doses. However, the variability in reported values across facilities was considerable, often exceeding a tenfold difference, and was even greater than that seen in radiopharmaceutical activities. In recent years, hybrid CT systems have increasingly incorporated automatic tube current modulation, and Artificial Intelligence-based image reconstruction using deep learning techniques is also being applied [14]. Naturally, substantial disparities in radiation dose are expected between institutions using cutting-edge imaging technology and those relying on equipment that is more than a decade old. Furthermore, differences in imaging protocols, such as the emphasis on image quality versus dose reduction and variations in scan coverage, likely contribute to the observed variation.

Although there are relatively few studies on radiation exposure from hybrid CT in the field of nuclear medicine, variations among countries in radiation exposure from hybrid CT have been reported. A meta-analysis of 27 studies including 12 on national DRLs reported CTDIvol and DLP values for hybrid CT in nuclear medicine [15]. For ^99m^Tc cardiac SPECT/CT, CTDIvol and DLP varied by factors of 2.1-fold (4.1 mGy in Japan [2] vs. 2.0 mGy in Switzerland [16]) and 2.4-fold (85 mGy·cm in Japan [2] vs. 36 mGy·cm in the UK [17]), respectively. For ^18^F-FDG whole-body PET/CT, the variation was even greater, at 3.2-fold for CTDIvol (13.07 mGy in New Zealand [18] vs. 4.1 mGy in Kuwait [19]) and 2.8-fold for DLP (1319 mGy·cm in New Zealand [18] vs. 474 mGy·cm in Australia [18]). The authors of the review attributed these differences to several factors, including differences in the purpose of the CT examination (e.g., attenuation correction vs. diagnostic imaging), the type of IEC phantom used for CTDIvol calculation (e.g., a diameter of 16 cm vs. 32 cm), and inconsistent definitions of scan range (e.g., vertex to toes vs. skull base to mid-thigh for “whole-body” imaging). As with radiopharmaceuticals, these findings underscore the need for the harmonization and standardization of international imaging protocols.

## Limitations

There are several limitations to this study. First, the collected data showed substantial variation, and a considerable number of outliers were observed. Considering the data collection method, in which each facility independently submitted values via an online system, some variability was anticipated. Input errors, such as misplaced decimal points or incorrect units, appeared to be relatively common, and may have had some effect on data reliability. Future surveys may benefit from implementing automated validation rules and input guidance systems to reduce such errors. Second, several technical factors known to substantially affect radiation dose are not included in the survey items. These include the presence or absence of advanced imaging technologies, such as semiconductor detectors and time-of-flight functions in the scanners used. Furthermore, the effect of patient weight, which is an important factor affecting both the administered dose and image quality [19], was not considered. While collecting such detailed information from a large number of facilities may be challenging, it may be necessary to divide the investigation into several parts, such as SPECT and PET, radiopharmaceuticals, and hybrid CT. Third, although DRLs for radiological procedures including nuclear medicine are typically defined as just the 75th percentile of the collected data, the DRLs for nuclear medicine in this study were determined through a more qualitative approach. This included discussions among experts, placing particular emphasis on balancing diagnostic image quality with radiation dose. Although this method aimed to more accurately reflect clinical practices in Japan, it may have resulted in DRL values with different characteristics to those established in other countries. Other unaddressed factors include differences in imaging protocols among facilities, scan coverage, reconstruction algorithms, and the diversity of equipment models and manufacturers. These factors may have also affected the values of the obtained data. These limitations highlight the need for a more standardized data collection framework, and the inclusion of metadata in future DRL surveys, to enable more detailed analyses and to facilitate international comparisons.

## Conclusion

In this study, we established the Diagnostic Reference Levels 2025 (DRLs2025) by revising the previous DRLs for nuclear medicine procedures in Japan. Compared with previous surveys, moderate changes were observed; however, the overall downward trend in administered radiopharmaceutical activities and CT parameters of hybrid imaging suggests the continued progress in optimization of radiation exposure throughout Japan. The DRLs remain a key benchmark for promoting the optimization of radiation dose in clinical practice. This initiative represents a meaningful contribution to radiation protection and quality assurance in nuclear medicine in Japan. We aim to continue this effort to ensure sustained improvement and greater alignment with international standards.
